# Postgraduate medical education in obstetrics and gynaecology: Where are we now and what do we need for the future? A study on postgraduate training in obstetrics and gynaecology in Germany, Austria and Switzerland

**DOI:** 10.3205/zma001562

**Published:** 2022-09-15

**Authors:** Franziska M. Winder, Georg Breuer, Martine Favero, Philipp Foessleitner, Margareta Friemann, Benedict Krischer, Karin Windsperger, Martin Weiss

**Affiliations:** 1 Kantonsspital St. Gallen, Frauenklinik, St. Gallen, Switzerland; 2 Junges Forum der Schweizerischen Gesellschaft für Gynäkologie und Geburtshilfe – gynécologie suisse (SGGG), Bern, Switzerland; 3 Universitätsklinikum Tulln, Tulln an der Donau, Austria; 4 Junge Gyn in der Österreichischen Gesellschaft für Gynäkologie und Geburtshilfe (OEGGG), Wien, Austria; 5 Rhypraxis, Feuerthalen, Switzerland; 6 Medizinische Universität Wien, Universitätsklinik für Frauenheilkunde, Klinische Abteilung für Geburtshilfe und Feto-Maternale Medizin, Wien, Austria; 7 Universitätsspital Münster, Department für Gynäkologie und Geburtshilfe, Münster, Germany; 8 Junges Forum in der Deutschen Gesellschaft für Gynäkologie und Geburtshilfe (DGGG), Berlin, Germany; 9 Universität Zürich, Institut für Medizinische Genetik, Zürich, Switzerland; 10 Eberhard Karls Universität Tübingen, Department für Frauengesundheit, Tübingen, Germany

**Keywords:** clinical education, OBGYN-training, PGME, curriculum, international cooperation

## Abstract

**Objective:** In this study, we aim to assess the current situation of postgraduate medical education in obstetrics and gynaecology in Germany, Austria and Switzerland. In addition, we aim to determine transferable advantages amongst the countries.

**Study design: **We performed a survey through a digital questionnaire with a total of 40 questions. The survey was advertised via communication channels of the German, Austrian and Swiss gynaecological societies; the participants were enrolled anonymously.

**Results: **A total of 422 trainees took part in the survey. Differences within the three countries where found regarding the workload and the training of sub-specialties. Generally, the participants described to spend the majority of their daily working hours on documentation. Concerning assessment of current training regulations, more than half of trainees stated that they were actually faced with notable difficulties to fulfil the required obligatory numbers of self-performed interventions being documented. When asked for their intrinsic feeling of safety, around two-third of trainees felt “confident to very confident” during standard interventions. These numbers were up to 12% higher in the group of trainees who experienced simulation training during their education.

**Conclusion: **With the help of this survey, weak points can be identified such as workload and implementation of current training regulations. Projects and ideas as EBCOG PACT, EPAs, the reduction of bureaucracy through digitization and deepening skills through simulation make a valuable contribution to compensate for these deficits and to adapt to future requirements.

## Zusammenfassung

**Ziel: **Diese Studie stellt die aktuelle Situation der medizinischen Weiterbildung zum Facharzt für Gynäkologie und Geburtshilfe in Deutschland, Österreich und der Schweiz dar. Zusätzlich werden adaptierbare Stärken der jeweiligen Länder identifiziert.

**Studiendesign: **Wir führten eine Online-Umfrage mit 30 Fragen durch. Die Umfrage wurde durch verschiedenste Kommunikationskanäle der Deutschen, Österreichischen und Schweizer Gesellschaft für Gynäkologie und Geburtshilfe beworben. Die Teilnahme war freiwillig und anonym.

**Resultat:** 422 Assistenzärzt*innen und junge Fachärzt*innen nahmen an der Umfrage teil. Unterschiede zwischen den drei Ländern zeigten sich bei den Aufgaben der Ärzt*innen und dem Training in Sub-Spezialisierungen. Grundsätzlich berichteten die Teilnehmer*innen, dass sie einen Großteil ihrer Arbeitszeit mit Dokumentation verbringen. Ein beunruhigendes Ergebnis ist die Tatsache, dass mehr als die Hälfte der Teilnehmer*innen angaben, Schwierigkeiten mit der Erfüllung der geforderten Fallzahlen für selbstdurchgeführte Eingriffe zu haben. Auf die Frage, wie sicher sie sich bei Standardeingriffen fühlen, gaben 2/3 an, „sich sicher bis sehr sicher“ zu fühlen. Diese Zahl war 12% höher bei Assistenzärzt*innen, welche während ihrer Weiterbildung Simulationstrainings besucht haben.

**Zusammenfassung: **Mit Hilfe dieser Umfrage konnten Schwachstellen bei der Arbeitsverteilung und der Umsetzung aktueller Weiterbildungsinhalte identifiziert werden. Projekte und Ideen wie EBCOG PACT und EPA, die Reduktion von Bürokratie, und die Vertiefung von Fähigkeiten durch Simulationstraining helfen individuelle Schwachstellen zu kompensieren und für die Zukunft vorbereitet zu sein.

## Introduction

High quality training is essential to become a specialist in obstetrics and gynaecology (OBGYN) and to ensure optimal patient care in the future. Every country in Europe defined specific goals and qualifications of training for graduate and postgraduate medical education (PGME) [1]. Being individually defined and implemented in the countries, the curricula considerably differ in most parts of Europe, e.g. in the catalogue of requirements. Differences in training are often caused by differing medical infrastructure of most countries, diverging clinical responsibilities of subspecialties and the lack of protected timeslots for practical and theoretical training. For example, breast surgery which is mandatory content of training curricula in some countries, is not even being trained in others. In 2009, Rodriguez et al. found major differences in definition of training content and outcome among European trainees in OBGYN of every level of experience [2]. The resulting need for harmonization of European training outcomes is reflected by the establishment of the pan-European curriculum for training in OBGYN by the European Board and College of Obstetrics and Gynaecology (EBCOG). 

The neighbouring German-speaking countries Germany, Austria and Switzerland vary substantially regarding their training curricula; however, they are highly comparable regarding medical infrastructure and health care systems. Therefore, these countries are ideal models to study the effects of different training curricula on educational outcomes [1] and satisfaction of trainees. In this study, we aim to provide a representative picture of the current situation of training in OBGYN in Germany, Austria and Switzerland. Furthermore, we intend to determine transferable advantages of the different systems. 

## Methodology

We considered a survey to be the most appropriate method to get a comprehensive overview of the general situation and satisfaction of trainees, since it allows a large group of people to participate, even in case of limited time and financial resources and even if they are geographically diversified. Participants were enrolled anonymously. We performed the survey through a digital questionnaire with a total of 30 questions (see attachment 1 ). The questionnaire was designed by consensus of experts, all board members of a cooperation, including representatives of trainee networks of the German (DGGG), Swiss (SGGG) and Austrian (OEGGG) society for obstetrics and gynaecology. Advice was given by eight gynaecologists (FMW, KW, GB, MF, PF, MF, BK, MW) from Germany, Austria and Switzerland, who are partially involved in the development of national OBGYN curricula. The experts chose to focus on certain topics (such as simulation programs, training regulations or training in subspecialties) in order to provide a representative picture of the current situation of trainees but prevent a question overload of the survey. The questionnaire was set-up in German as online questionnaire allowing wide accessibility. The questions were presented sequentially. A new question was shown only if the preceding was answered. Thus, for completion of the survey the respondent had to answer all 30 questions. The survey was controlled and circulated by the Swiss Federal Institute of Technology in Zurich (ETH), Department for Health Sciences and Technology, Consumer Behavior and was advertised via communication channels of the societies. Data collection was performed from August to September 2020. Multiple participations by one person were excluded by an anonymous IP address check. The evaluation of data was carried out with SPSS 26.0 (IBM Corp.).

## Results

### Characteristics of participants

A total of 422 trainees took part in the survey. Of these 209 (49.5%) were currently trained in Germany, 116 (27.5%) in Switzerland and 97 (23%) in Austria. 88.9% were female and the median age was 32 years. Around three-fourths of the study participants were in the third year of training or more. 42% worked at a large hospital and training facility with at least 500 beds. 77% of participants worked full-time. 

#### Part-time work and work sharing

Although the largest number of participants worked full time, the majority (70%) rated part-time workloads between 80 and 95 % as the most attractive employment obligation. However, this did not seem to reflect a general desire to work less, as part-time work with less than 40% pensum was classified as “not attractive or not at all attractive” by 82% and 40-55% pensum were perceived as considerably less attractive by 45% of participants. Overall, part-time working models seemed to be widespread already, as 94% of the participants confirmed that their hospital offered some form of reduced pensum (see table 1 (Tab. 1)).

#### Workload 

**Figure 1:**
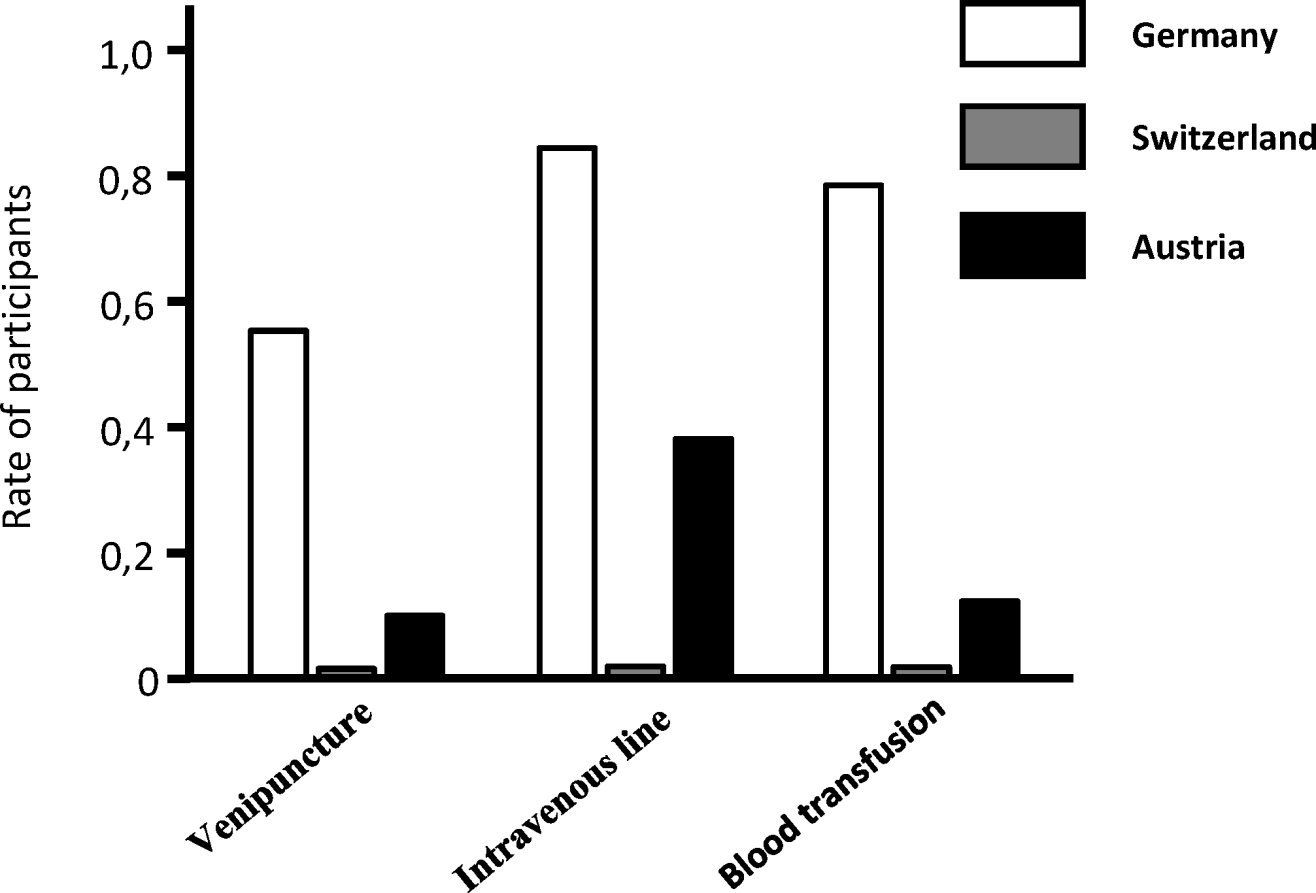
Routine clinical work carried out by doctors.

Notably, we found distinct country-specific differences in the performance of medical procedures by non-medical healthcare professionals. Certain invasive interventions no longer need to be carried out by doctors only (see figure 1 (Fig. 1)). For example, 98% of the trainees in Switzerland stated that they “never or rarely” insert an intravenous line, whereas 85% of German trainees “always to often” performed this intervention. Generally, the trainees described to spend the majority of their daily working hours on documentation. 27% perceived this as “not or not at all efficient” and only a quarter of the trainees indicated to have an assistant available (e.g. ward assistant), supporting them in documentation and organization tasks.

#### Training of sub-specialties in OBGYN

**Figure 2:**
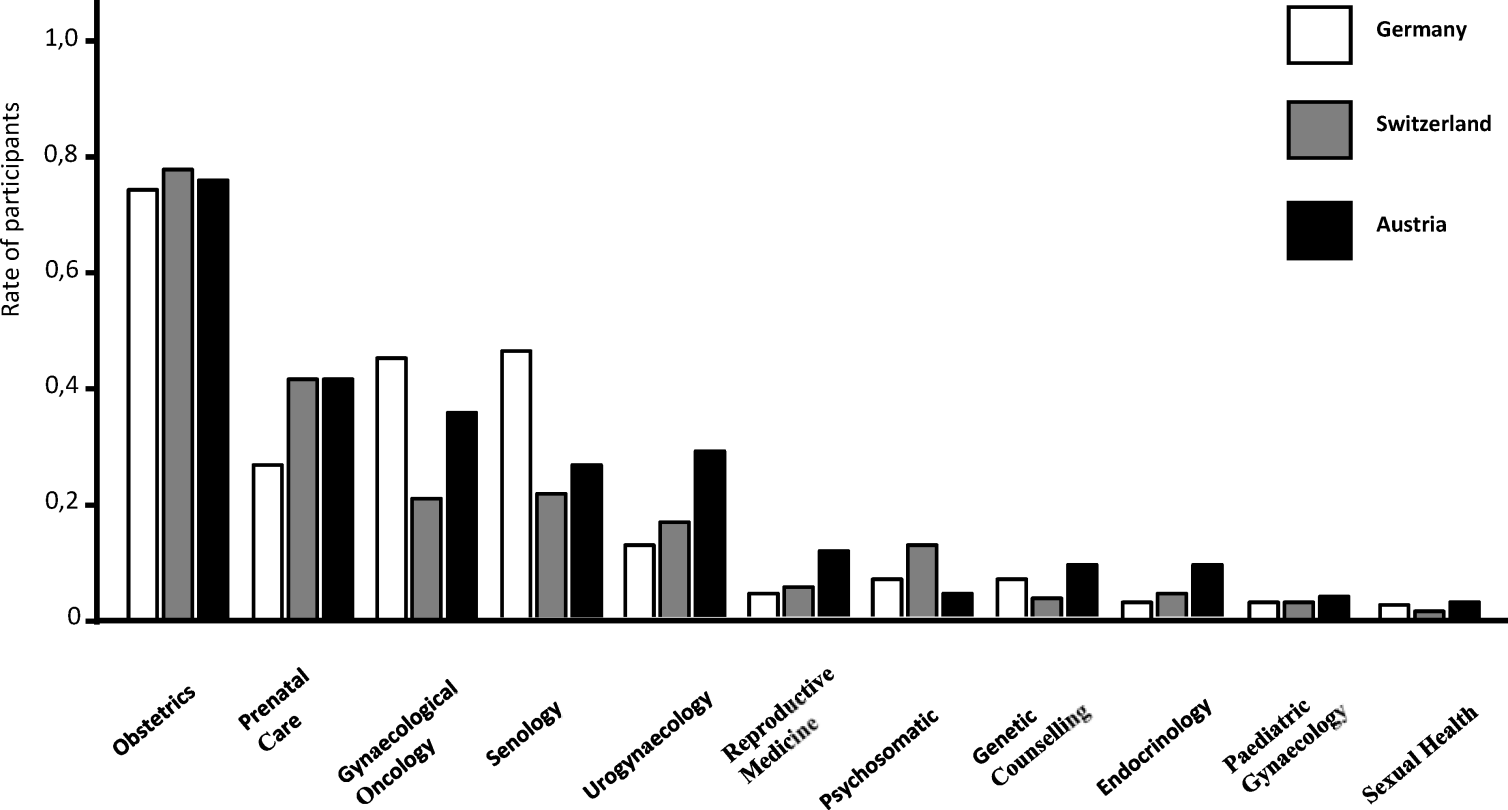
Sub-specialities rated as “well” and “very well” represented in PGME in OBGYN in Germany, Austria and Switzerland

Regarding the training of specific sub-areas in OBGYN, 76% of participants rated obstetrics as “well to very well” represented in all participating countries (see figure 2 (Fig. 2)). Major differences between the countries were observed in gynaecological oncology and senology as well as in prenatal care. Interestingly, only 5% of participants stated Endocrinology being “well or very well” covered within their curricula. Paediatric Gynaecology, as well as Sexual Health and Reproductive Medicine were seen as underrepresented sub-areas in OBGYN training by a vast majority of participants (see attachment 2 ).

#### Assessment and implementation of current training regulations

**Figure 3:**
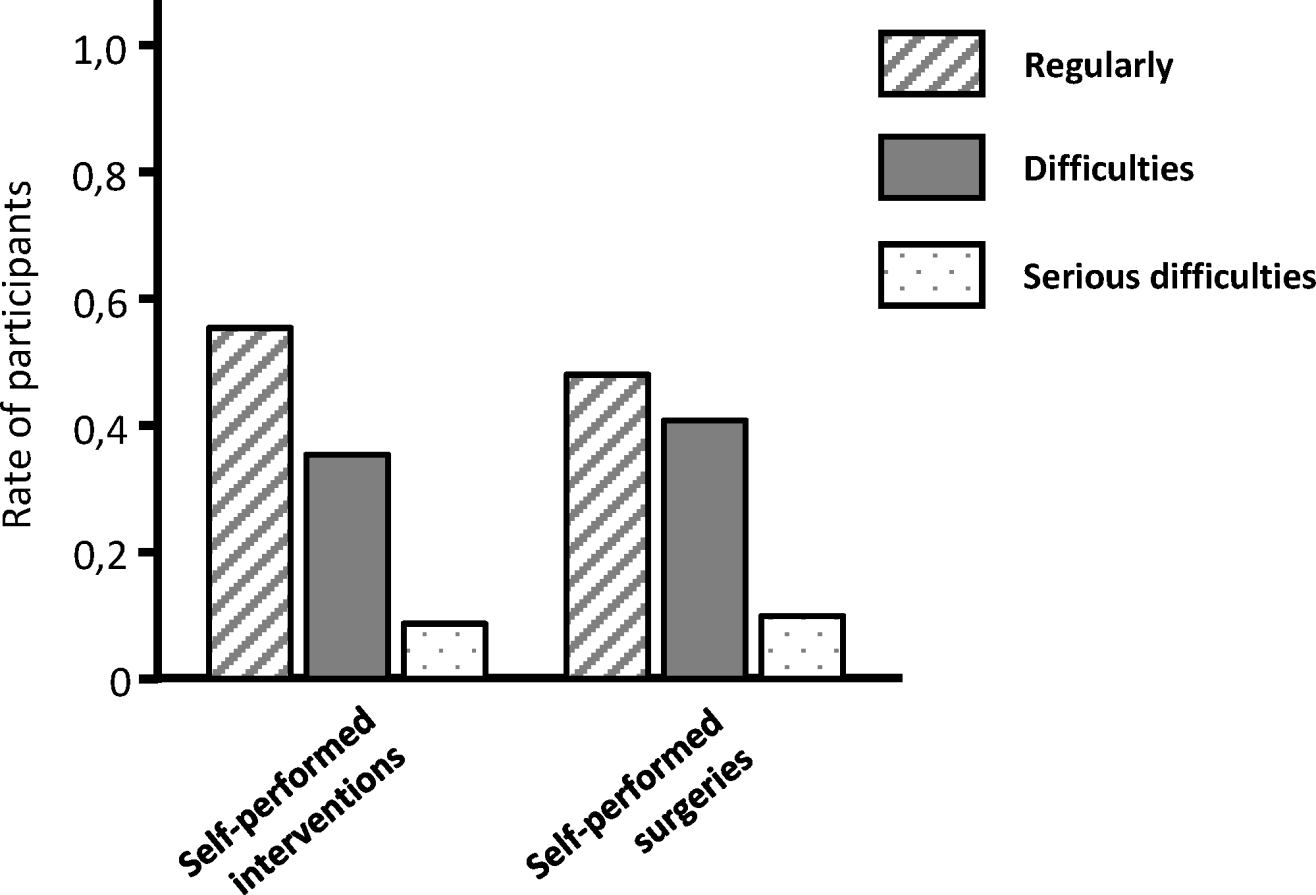
Fulfilment-rate of self-performed interventions being obligatory for PGME

Interestingly, only 22% of participants felt “well to very well” prepared for their work as a specialist in the hospital setting and only 11% felt “well to very well” prepared for working in private practice. The majority of trainees assessed themselves only “moderately prepared” for the further work in a hospital (66%) or private practice (57%). In addition, this was associated with the fact that only 47% of trainees stated that they regularly fulfilled the required obligatory numbers of self-performed interventions (see figure 3 (Fig. 3)). 53% of participants stated that they were actually faced with notable to serious difficulties to fulfil the required obligatory numbers of self-performed interventions being documented. 

As a result, two-thirds of the participants agreed that an electronically kept logbook is useful when documenting the obligatory interventions. However, the survey showed substantial, country-specific differences in the actual implementation of an electronically kept logbook. Whereas 86% of the participants from Switzerland answered that their logbook is kept electronically, only 5% from Germany and 1% from Austria currently kept their logbook electronically. 

Furthermore, an annual evaluation interview being documented in written form was offered to only 48% of participants. 54% of the trainees stated that they had a supervisor giving advice on questions with medical content or about career planning.

#### Confidence during intervention

To determine if the lack of self-performed interventions and the corresponding documentation have an impact on the feeling of security among the trainees, participants were asked how confident they feel in standard situations and interventions in OBGYN. Thereby, the intrinsic feeling of safety when they perform standard surgery is an important parameter for determining the quality of training. Interestingly, around two-thirds of trainees felt “confident to very confident” during standard interventions like curettage, Caesarean section and hysteroscopy. Among other interventions like simple laparoscopy and vacuum extraction as well as the management of emergencies in obstetrics like postpartum bleeding, or shoulder dystocia more than half of participants felt only "moderately" confident. When it comes to rare situations like breech birth or forceps delivery, most of the participants felt “not” or “not at all” confident (see attachment 3 ).

Considering the years of specialty training, the feeling of security among frequently performed interventions such as the curettage, hysteroscopy and Caesarean section increased over time.

**Figure 4:**
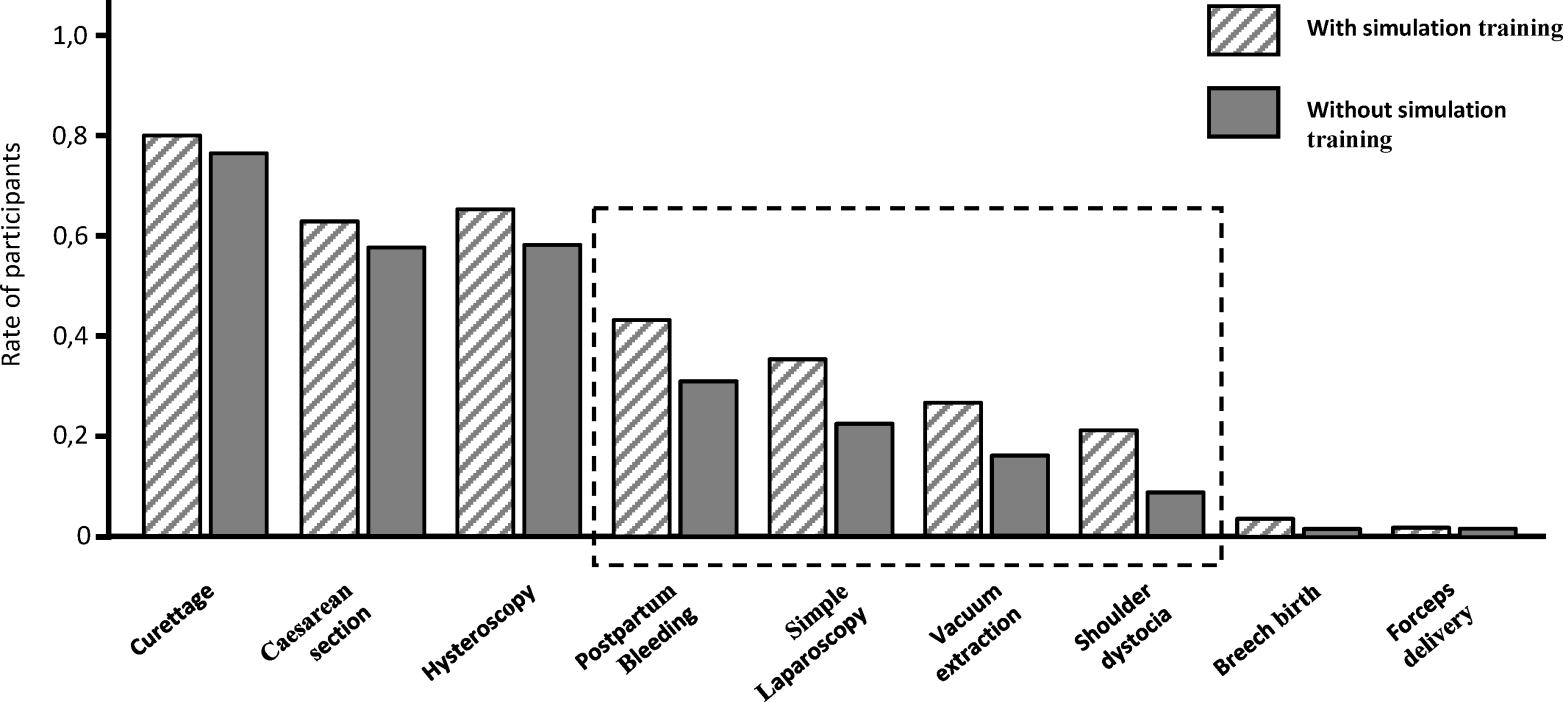
The impact of simulation training on intrinsic feeling of safety among trainees during standard situations and intervention

However, divided into groups with and without a simulation training in obstetrics (44% with simulation training) or gynaecology (20%) offered in their hospital there was a noticeable increase of safety among the trainees who could use simulation training for their further education. Particularly, in these interventions (simple laparoscopy, management of postpartum bleeding or shoulder dystocia and vacuum extraction) that can arise in a hospital at any time, the trainees with simulation training feel up to 12% more confident than those participants without (see figure 4 (Fig. 4); box with broken line: noticeable differences between “with” and “without” simulation training).

## Discussion

This study aimed to identify the current situation of OBGYN training as well as transferable advantages of the different training systems in Germany, Austria and Switzerland. Anonymous surveys on “customer satisfaction” with their training and work situation, but also the assessment of the heads of the facilities are centrally recorded in some countries. Since 1996, the Swiss Institute for Medical Training (SIWF) has been carried out an annual survey among Swiss trainees which has served as a model for the current international survey. For decades, the results of the SIWF survey have served as feedback in order to recognize and promote successful concepts or to promptly uncover weak issues. Annually, the results of the survey are published online, and thus, offer young doctors an assessment basis for choosing an attractive employment and training position. At the same time, the data provide an annual benchmarking of the institutional training quality [3]. Cross-border cooperation offers a great opportunity to learn and benefit from other training systems. PGME is teamwork that requires shared commitment to innovation, shared responsibility, supportive frameworks, and a teaching culture [4]. Besides a few regional and interdisciplinary evaluation projects [5], [6], to our best knowledge this survey is the first cross-border project with special focus on OBGYN as specific subject area. This data serves as a valuable basis for further research and development in the field of supranational PGME in OBGYN.

To overcome country-specific differences in training there has been great effort to harmonize training standards in OBGYN. The tendency of pan-European harmonization is the result of the increasing mobility of medical specialists and patients and the need for quality assurance of training throughout Europe [7], [8], [9]. However, within the European Union, all countries have mutually recognized training qualifications for graduate and postgraduate medical education. This mutual recognition mostly is not content-related but based on minimum requirements, including training sites (recognized teaching hospitals) and duration of training [1], [10], [11]. A push in the direction of a common, harmonized, European curriculum for advanced training in OBGYN is the Project for Achieving Consensus in Training (PACT) of the European Board and College of Obstetrics and Gynaecology [12]. The curriculum defines content and competencies during a three-year basic training course (so-called “core”), which is the same for every gynaecologist. This is followed by a two-year advanced training phase with elective modules that can be chosen depending on the desired profile (so-called “electives”). Therefore, the EBCOG PACT offers sufficient opportunities to overcome country-specific burdens of PGME. Within our study group, Austria has already implemented the EBCOG PACT structure in OBGYN PGME. 

An important step towards improvement and maintenance of high quality PGME is to distribute the limited time resources as best as possible and to restructure non-medical tasks or to evaluate bureaucratic processes [6]. According to a recent study by Trezzini et al., a trainee spends 167 minutes per day on documenting patient records. This corresponds to 27% of their working time. Instead of reduction, the medical bureaucracy has substantially increased in recent years [13]. Also, within the present survey, the participants complained that bureaucracy takes up a large part of their everyday working time. Medical documentation and organization of standard procedures are seen to be inefficient and do drastically reduce the satisfaction of trainees [13]. If additional tasks as venipuncture or insertion of an intravenous line are performed by trainees, this has major impact on patient care and quality of PGME. Simple tools such as digitized Dictaphones with voice recognition, but also major structural changes such as clinical nurses or physician assistants assuming tasks of clinical routine could considerably relieve the workload and increase time dedicated to PGME.

In light of increasing workload and bureaucracy, it is not surprising that required obligatory numbers of interventions can barely be fulfilled during the standard length of training. However, instead of adjusting numbers or structure, the results suggest that missing interventions were subsequently attested and documented. Are the required numbers of different interventions too high for the existing number of cases? Can certain interventions and diagnostic measures only be carried out in specialized centres? Is the current routine clinical workload and bureaucratic effort incomparable to former generations of trainees? Comparing the logbooks of the three countries regarding the required number of interventions and diagnostic measures, serious differences are detectable. Whereas in Austria and Switzerland a total of 85 and 80 obstetric interventions performed by the trainee are required, respectively, German trainees only need 25 Caesarean sections and “contribution” in further obstetric interventions. 50 colposcopies are required in Germany and Switzerland, however, only 20 in Austria. A total of 275 gynaecological surgeries are required in Austria, 255 in Switzerland, and only 200 in Germany.

We have to strike out new paths in order to ensure that not numbers, but practical verifications attest the level of training in OBGYN. So-called “Entrustable Professional Activities” (EPAs) can support the relationship between trainer and trainee being part of competence-based medical education. An EPA is a detailed description of a medical activity, e.g. a Caesarean section, which combines the knowledge, skills and attitudes required for this procedure. In countries such as the Netherlands and Canada, EPAs have already found their way into continuing medical education in various specialties ([14] Einleitung, [15]. They support the change in PGME, away from an “on-off knowledge-based” examination at the end of training to a modern, practice-adapted and competence-oriented training concept. Trainees receive timely feedback on their activities and annual goals can be defined and evaluated. EPAs thus also form a valuable basis for annual evaluation meetings. An electronically kept logbook is also indispensable for recording such advanced training competencies and target-oriented evaluation discussions. In addition to written documentation, the digital form also enables a timely evaluation of the level of training and should be an essential part of a modern training program.

Simulation training covers a wide range of training opportunities from high-tech team simulation training and skill drills to low fidelity training units. Each of these methods has its justification, as they train completely different abilities. While the team simulation training, which is increasingly established in obstetrics, is primarily about consolidation and training of treatment coordination, low fidelity models help to understand concepts based on simple technical repetitions. Although, simulation units are often accompanied by high costs, the present survey illustrates the positive impact of this additional training. In our survey, trainees who confirmed participation in any type of simulation training felt more confident especially in situations and interventions that are part of the basic training of OBGYN such as simple laparoscopy or postpartum bleeding. Simulation training offers a great benefit for modern PGME by increasing the efficiency in gaining experience and thus, improving the patients' safety. 

Like the SIWF survey, this study can not cover all aspects of the current training situation in the three countries. The study group chose to focus on certain topics that shape the daily worklife of trainees. The selection of topics and data was done to our best knowledge but is a limitation to the study. Certainly, further studies that cover more aspects of the basic training in OBGYN are needed to create a broader picture of the current situation of training in OBGYN in Germany, Austria and Switzerland.

## Conclusion

The current postgraduate training for OBGYN is already at a very high level in Germany, Austria and Switzerland. The aim is to jointly further develop this advanced training to be future-oriented. With the help of this survey, current weak points can be identified. Projects and ideas such as EBCOG PACT, EPAs, the reduction of bureaucracy through digitization and deepening skills through simulation training make a valuable contribution to compensate for these deficits and to adapt to future requirements. In this way, it is possible to secure the high level of European postgraduate training in OBGYN for future generations.

## Acknowledgements

We thank Larissa Luchsinger and Jeanine Ammann, research assistant at ETH Zurich, for the helpful evaluations, discussions and additions to this survey.

We thank everyone who participated in the study. The study was funded with support of the DGGG, OEGGG and SGGG, however, the societies had no further involvement except for the financial support. 

## Competing interests

The authors declare that they have no competing interests. 

## Supplementary Material

Supplementary material – original ouestionnaire

Sub-specialties represented in PGME in OBGYN in Germany, Austria and Switzerland

Intrinsic feeling of safety among trainees during standard situations and interventions

## Figures and Tables

**Table 1:**
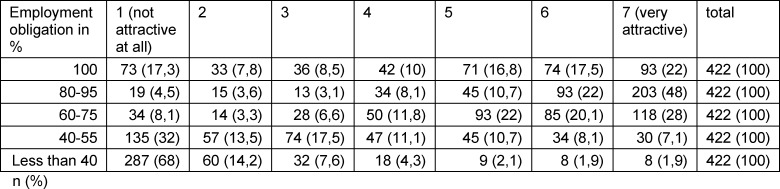
Attractiveness of employment obligations in OBGYN

## References

[1] Council of the European Union (2005). Directive 2005/36/EC of the European Parliament and of the Council of 7 Sep 2005 on the recognition of professional qualifications. Off J Eur Union.

[2] Rodríguez D, Christopoulos P, Martins N, Pärgmäe P, Werner HM (2009). Working conditions survey and trainees situation: New approach to auditing the situation of European trainees in obstetrics and gynaecology ten years later. Eur J Obstet Gynecol Reprod Biol.

[3] Sütterlin B, Burgermeister L, Siegrist M, Bauer W (2017). Erfreulich hoch eingeschätzt: Der Stellenwert der Weiterbildung an den Spitälern. Schweiz Ärzteztg.

[4] Bank L, Jippes M, van Rossum TR, den Rooyen C, Scherpbier AJ, Scheele F (2019). How clinical teaching teams deal with educational change: ‘We just do it’. BMC Med Educ.

[5] Vogel S, Leffmann C, Bruns-Matthiessen B, Orlow P, Siegrist M, Krystofiak T, Fotuhi P (2009). Evaluation der Weiterbildung: Gute Weiterbildung ist kein Zufall. Dtsch Arztebl.

[6] Hilienhoff A, Osterloh F (2019). Assistenzärzte: Zu wenig Zeit für die Patienten. Dtsch Arztebl Int.

[7] García-Pérez MA, Amaya C, Otero Á (2007). Physicians’ migration in Europe: An overview of the current situation. BMC Health Serv Res.

[8] Herfs PG (2014). Aspects of medical migration with particular reference to the United Kingdom and the Netherlands. Hum Resour Health.

[9] Forcier MB, Simoens S, Giuffrida A (2004). Impact, regulation and health policy implications of physician migration in OECD countries. Hum Resour Health.

[10] Peeters M (2007). Free movement of medical doctors in the EU. Med Law.

[11] Costigliola V (2011). Mobility of medical doctors in cross-border healthcare. EPMA J.

[12] Scheele F, Novak Z, Vetter K, Caccia N, Goverde A (2014). Obstetrics and gynaecology training in Europe needs a next step. Eur J Obstet Gynecol Reprod Biol.

[13] Trezzini B, Meyer B, Ivankovic M, Jans C, Golder L (2020). Der administrative Aufwand der Ärzteschaft nimmt weiter zu. Schweiz Ärzteztg.

[14] Breckwoldt J, Beckers S, Breuer G, Marty A (2018). "Entrustable professional activities". Zukunftsweisendes Konzept für die ärztliche Weiterbildung. Anaesthesist.

[15] ten Cate O (2006). Trust, competence, and the supervisor’s role in postgraduate training. Br Med J.

