# Supplementary material for: Postgraduate medical education in obstetrics and gynaecology: Where are we now and what do we need for the future? A study on postgraduate training in obstetrics and gynaecology in Germany, Austria and Switzerland
Source: GMS J Med Educ. 2022 Sep 15;39(4):Doc41. doi: 10.3205/zma001562 (PMC9585411; doi:10.3205/zma001562)
Supplement: Supplementary material – original ouestionnaire [file JME-39-41-s-001.pdf]

Attachment 1: Supplementary material - original ouestionnaire

Part 1 (personal information)

1. In which country are you working as a doctor?
   a. Switzerland
   b. Germany
   c. Austria

2. Please enter your gender:
   a. Male
   b. Female
   c. Divers

3. Please enter your birthyear:

4. In which year did you finish Medical School?

5. How far in to your training are you?
   a. ...years
   b. I`m finished.

6. Have you already identified a field of specialisation (e.g. gyn. Oncology, Obstetrics, fetomaternal Medicine) for your further career?
   a. Yes
   b. no

7. For how many months have you been working at your current hospital?

8. How big is your current hospital according to the bed count?
   a. Up to 200 beds
   b. 201 to 499 beds
   c. 500 and more beds

9. What is your current employment obligation?
   a. 100%
   b. 80-95%
   c. 60-75%
   d. 40-55%
   e. Less than 40%

10. How happy are you with your current employment obligation?
1 = very happy / 7 = not happy at all

11. How many hours are you working on average in a week?

12. Does you hospital offer part-time jobs?
a. Yes
b. No
c. Don`t know

13. In your opinion, how attractive are following part-time employment obligation? (1= not attractive at all, 7= very attractive)
a. 100%
b. 80-95%
c. 60-75%
d. 40-55%
e. Less than 40%

<u>Part 2 (questions concerning your training)</u>

1. Imagine following intervention or situation. How confident do you feel performing the intervention or managing the situation on your own? (1= not at all; 7= very confident)
a. Forceps delivery
b. Vacuum extraction
c. Postpartum bleeding
d. Shoulder dystocia
e. Breech birth

2. Imagine following surgical intervention. How confident do you feel performing the intervention on your own? (1= not at all; 7= very confident)
a. Curettage
b. Hysteroscopy
c. Simple laparoscopy
d. Caesarean section

3. How well prepared do you feel to work as a specialist... (1= not at all, 7=very well prepared)
a. In a private practice
b. In a hospital setting

4. Does your hospital have a structured simulation training? (Yes/No/Don`t know)
a. For obstetrics (e.g. skill drills)?
b. For surgical gynecology
c. If yes: does it happen during working hours?

Attachment 1 to Winder FM, Breuer G, Favero M, Foessleitner P, Friemann M, Krischer B, Windsperger K, Weiss M. *Postgraduate medical education in obstetrics and gynaecology: Where are we now and what do we need for the future? A study on postgraduate training in obstetrics and gynaecology in Germany, Austria and Switzerland.* GMS J Med Educ. 2022;39(4):Doc41. DOI: 10.3205/zma001562

5. What do you think: how well are following topics covered during your specialisation? (1= not at all, 7=very well)
a. Endocrinology
b. Reproductive medicine
c. Urogynaecology
d. Gyn. Oncology
e. Senology
f. Sexual health
g. Psychosomatic
h. Paediatric gynaecology
i. Genetic counselling
j. Obstetrics
k. Prenatal care

6. Please let us know how much you agree with the following statements when you think about your training (1=not at all, 7= very much)
a. We have structured teaching (e.g. speeches, journal club,…)
b. We have a good “teaching culture” (bedside teaching)
c. We have enough number of cases
d. There is an arbitrariness of the superior (e.g. concerning rotation arrangement or surgical planning)
e. We have a structured rotation system.
f. The law on working hours is followed.
g. There is a lot of bureaucracy.
h. There is discrimination against trainees working part time.
i. I wish for more simulation training.

7. How often do you perform following routine clinical work?
a. Venipuncture
b. Blood transfusion
c. Intravenous line

8. Please let us know how much you agree with the following statements when you think about your training (1=not at all, 7=very much)
a. The surgical interventions listed in my logbook were performed by myself/as a “first surgeon”.
b. The non-surgical interventions (e.g. colposcopy, ultrasound,…) listed in my logbook were performed by myself.
c. My logbook is kept electronically.
d. I think an electronically kept logbook is good and reasonable.

9. Please let us know how your typical working day looks like:
   a. How much % of your daily working time do you spend with patients? …%
   b. How much % of your daily working time do you spend with administration and documentation? …%
   c. How much % of your daily working time do you spend with other/above not listed activities? …%

10. Do you have someone assisting you at documentation or organisation (e.g. documentation assistant)?
    a. Yes
    b. No

11. What do you think how efficient is your documentation? (1=not efficient at all, 7= very efficient)

12. Do you work with electronic patient files?
    a. Yes
    b. No

<u>Part 3 (questions concerning feedback culture and mentoring)</u>

1. Do you have a superior who you can contact in case of questions concerning your training?
   a. Yes
   b. No

2. Do you have a superior who you can contact concerning your career planning?
   a. Yes
   b. No

3. Do you know the "Project for Achieving Consensus in Training" of the European Board and College of Obstetrics and Gynaecology (EBCOG-PACT)?
   a. Yes, I`m familiar with it.
   b. Yes, I have heard of it.
   c. No, never heard of it.

4. Please let us know how much you agree with the following statements when you think about your training (1=not at all, 7=very much)

a. My superior has enough pedagogic competence to teach me.
b. I receive feedback about my work on a regular base.
c. My superior has enough time to explain complex topics to me and answer my questions.
d. My superior supports me in difficult decisions within a reasonable period.
e. In my hospital, we discuss our diagnostics and therapies on the basis of current literature and guidelines on a regular base.
f. My superiors demonstrate on a regular base that all aspects (social, mental and medical) need to be consider to make a decision.

We perform an annual evaluation interview which is documented in written form.
