# Supplementary material for: Postgraduate medical education in obstetrics and gynaecology: Where are we now and what do we need for the future? A study on postgraduate training in obstetrics and gynaecology in Germany, Austria and Switzerland
Source: GMS J Med Educ. 2022 Sep 15;39(4):Doc41. doi: 10.3205/zma001562 (PMC9585411; doi:10.3205/zma001562)
Supplement: Sub-specialties represented in PGME in OBGYN in Germany, Austria and Switzerland [file JME-39-41-s-002.pdf]

Attachment 2: Sub-specialties represented in PGME in OBGYN in Germany, Austria and Switzerland

		**1 (not at all)**	**2**	**3**	**4**	**5**	**6**	**7 (very well)**	**total**
**Obstetrics**	**Germany**	1 (0,5)	0 (0)	4 (1,9)	13 (6,3)	35 (16,9)	69 (33,3)	85 (41,1)	207 (100)
	**Switzerland**	0 (0)	0 (0)	2 (1,7)	1 (0,9)	22 (19,0)	42 (36,2)	49 (42,2)	116 (100)
	**Austria**	3 (3,1)	0 (0)	1 (1,0)	5 (5,2)	14 (14,4)	23 (23,7)	51 (52,6)	97 (100)
**Prenatal care**	**Germany**	16 (7,7)	19 (9,1)	35 (16,7)	46 (22)	38 (18,2)	22 (10,5)	33 (15,8)	209 (100)
	**Switzerland**	1 (0,9)	7 (6)	9 (7,8)	15 (12,9)	36 (31)	23 (19,8)	25 (21,6)	116 (100)
	**Austria**	6 (6,2)	4 (4,1)	9 (9,3)	17 (17,5)	21 (21,6)	19 (19,6)	21 (21,6)	97 (100)
**Gyn. Oncology**	**Germany**	6 (2,9)	11 (5,3)	20 (9,6)	31 (14,8)	48 (23)	50 (23,9)	43 (20,6)	209 (100)
	**Switzerland**	4 (3,4)	9 (7,8)	14 (12,1)	32 (27,6)	32 (27,6)	16 (13,8	9 (7,8)	116 (100)
	**Austria**	3 (3,1)	6 (6,2)	10 (10,3)	15 (15,5)	28 (28,9)	19 (19,6)	16 (16,5)	97 (100)
**Senology**	**Germany**	17 (8,1)	16 (7,7)	20 (9,6)	27 (12,9)	35 (16,7)	50 (23,9)	44 (21,1)	209 (100)
	**Switzerland**	4 (3,4)	14 (12,7)	12 (10,3)	30 (25,9)	30 (25,9)	18 (15,5)	8 (6,9)	116 (100)
	**Austria**	22 (22,7)	8 (8,2)	11 (11,3)	17 (17,5)	13 (13,4)	14 (14,4)	12 (12,4)	97 (100)
**Urogynaecology**	**Germany**	31 (14,8)	41 (19,6)	34 (16,3)	42 (20,1)	37 (17,7)	13 (6,2)	11 (5,3)	209 (100)
	**Switzerland**	2 (1,7)	13 (11,2)	23 (19,8)	20 (17,2)	39 (33,6)	15 (12,9)	4 (3,4)	116 (100)
	**Austria**	5 (5,2)	16 (16,5)	19 (19,6)	12 (12,4)	17 (17,5)	16 (16,5)	12 (12,4)	97 (100)
**Reproductive Medicine**	**Germany**	136 (60,3)	45 (21,5)	21 (10)	6 (2,9)	2 (1)	3 (1,4)	6 (2,9)	209 (100)
	**Switzerland**	27 (23,3)	34 (29,3)	21 (18,1)	16 (13,8)	12 (10,3)	4 (3,4)	2 (1,7)	116 (100)
	**Austria**	40 (41,2)	22 (22,7)	6 (6,2)	11 (11,3)	7 (7,2)	4 (4,1)	7 (7,2)	97 (100)
**Psychosomatic**	**Germany**	75 (35,9)	51 (24,4)	31 (14,8)	29 (12,9)	11 (5,3)	10 (4,8)	4 (1,9)	209 (100)
	**Switzerland**	22 (19)	28 (24,1)	22 (19)	15 (12,9)	15 (12,9)	10 (8,6)	4 (3,4)	116 (100)
	**Austria**	57 (59,4)	21 (21,9)	7 (7,3)	7 (7,3)	1 (1)	3 (3,1)	0 (0)	96 (100)
**Genetic Counselling**	**Germany**	93 (44,5)	43 (20,6)	28 (13,4)	14 (6,7)	16 (7,7)	6 (2,9)	9 (4,3)	209 (100)
	**Switzerland**	36 (31)	36 (31)	21 (18,1)	13 (11,2)	6 (5,2)	4 (3,4)	0 (0)	116 (100)
	**Austria**	38 (39,2)	21 (21,6)	10 (10,3)	13 (13,4)	6 (6,2)	6 (6,2)	3 (3,1)	97 (100)
**Endocrinology**	**Germany**	116 (55,5)	64 (30,6)	17 (8,1)	4 (1,9)	4 (1,9)	1 (0,5)	3 (1,4)	209 (100)
	**Switzerland**	16 (13,8)	29 (25)	35 (30,2)	15 (12,9)	16 (13,8)	4 (3,4)	1 (0,9)	116 (100)
	**Austria**	32 (33)	24 (24,7)	11 (11,3)	16 (16,5)	5 (5,2)	4 (4,1)	5 (5,2)	97 (100)
**Paediatric Gynaecology**	**Germany**	105 (50,5)	52 (25)	29 (13,9)	12(5,8)	7 (3,4	1 (0,5)	2 (1)	208 (100)
	**Switzerland**	48 (41,7)	26 (22,6)	30 (26,1)	7 (6,1)	4 (3,5)	0 (0)	0 (0)	115 (100)
	**Austria**	38 (39,2)	28 (28,9)	17 (17,5)	5 (5,2)	6 (6,2)	1 (1)	2 (2,1)	97 (100)
**Sexual Health**	**Germany**	130 (62,2)	51 (24,4)	17 (8,1)	6 (2,9)	4 (1,9)	0 (0)	1 (0,5)	209 (100)
	**Switzerland**	44 (38,3)	44 (38,3)	14 (12,2)	8 (7)	3 (2,6)	2 (1,7)	0 (0)	115 (100)
	**Austria**	59 (60,8)	18 (18,6)	9 (9,3)	8 (8,2)	0 (0)	3 (3,1)	0 (0)	97 (100)

n (%)

Attachment 2 to Winder FM, Breuer G, Favero M, Foessleitner P, Friemann M, Krischer B, Windsperger K, Weiss M. *Postgraduate medical education in obstetrics and gynaecology: Where are we now and what do we need for the future? A study on postgraduate training in obstetrics and gynaecology in Germany, Austria and Switzerland.* GMS J Med Educ. 2022;39(4):Doc41. DOI: 10.3205/zma001562
