# Supplementary material for: Postgraduate medical education in obstetrics and gynaecology: Where are we now and what do we need for the future? A study on postgraduate training in obstetrics and gynaecology in Germany, Austria and Switzerland
Source: GMS J Med Educ. 2022 Sep 15;39(4):Doc41. doi: 10.3205/zma001562 (PMC9585411; doi:10.3205/zma001562)
Supplement: Intrinsic feeling of safety among trainees during standard situations and interventions [file JME-39-41-s-003.pdf]

Attachment 3: Intrinsic feeling of safety among trainees during standard situations and interventions

	1 (not at all)	2	3	4	5	6	7 (very confident)	total
Curettage	10 (2,4)	7 (1,7)	12 (2,8)	18 (4,3)	55 (13)	139 (32,9)	181 (42,9)	422 (100)
Caesarean section	15 (3,6)	13 (3,1)	14 (3,3)	35 (8,3)	88 (20,9)	113 (26,8)	144 (34,1)	422 (100)
Hysteroscopy	22 (5,2)	15 (3,6)	15 (3,6)	44 (10,4)	77 (18,2)	115 (27,3)	134 (31,8)	422 (100)
Postpartum bleeding	13 (3,1)	21 (5)	39 (9,2)	70 (16,6)	127 (30,1)	114 (27)	38 (9)	422 (100)
Simple Laparoscopy	53 (12,6)	46 (10,9)	55 (13)	76 (18)	87 (20,6)	54 (12,8)	51 (12,1)	422 (100)
Vacuum extraction	89 (21,1)	35 (8,3)	69 (16,4)	62 (14,7)	80 (19)	68 (16,1)	19 (4,5)	422 (100)
Shoulder dystocia	33 (7,8)	53 (12,6)	66 (15,6)	110 (26,1)	99 (23,5)	42 (10)	19 (4,5)	422 (100)
Breech birth	233 (55,2)	72 (17,1)	63 (14,9)	30 (7,1)	17 (4)	5 (1,2)	2 (0,5)	422 (100)
Forceps delivery	383 (90,8)	23 (5,5)	6 (1,4)	5 (1,2)	3 (0,7)	0 (0)	2 (0,5)	422 (100)

n (%)

Attachment 3 to Winder FM, Breuer G, Favero M, Foessleitner P, Friemann M, Krischer B, Windsperger K, Weiss M. *Postgraduate medical education in obstetrics and gynaecology: Where are we now and what do we need for the future? A study on postgraduate training in obstetrics and gynaecology in Germany, Austria and Switzerland.* GMS J Med Educ. 2022;39(4):Doc41. DOI: 10.3205/zma001562
